# Clinical Trends and Outcomes in Technology-Assisted Total Hip Arthroplasty

**DOI:** 10.3390/jcm13206035

**Published:** 2024-10-10

**Authors:** Konstantinos Oikonomou, Nicholas R. Kiritsis, Haleigh M. Hopper, James R. Satalich, Conor N. O’Neill, Brady Ernst, Jibanananda Satpathy

**Affiliations:** 1 School of Medicine, Virginia Commonwealth University, 1000 E Marshall St., Richmond, VA 23298, USA; haleigh.hopper@vcuhealth.org; 2 School of Medicine, Wake Forest University, 1 Medical Center Blvd., Winston-Salem, NC 27157, USA; nkiritsi@wakehealth.edu; 3 Department of Orthopaedic Surgery, Virginia Commonwealth University Medical Center, 1200 E Broad St., 9th Floor, P.O. Box 980153, Richmond, VA 23298, USA or satalich18@gmail.com (J.R.S.); brady.ernst@vcuhealth.org (B.E.); jibanananda.satpathy@vcuhealth.org (J.S.); 4 Department of Orthopaedic Surgery, Duke University Health System, 2301 Erwin Rd., Durham, NC 27710, USA; conornoneill@gmail.com

**Keywords:** arthroplasty, technology-assisted, robotics, THA

## Abstract

**Background/Objectives:** In recent years, there has been a widespread focus on implementing technology in total hip arthroplasty (THA) to further improve precision and outcomes. This study aimed to identify recent trends in the utilization, clinical variables, and rate of adverse events for technology-assisted THA (TA-THA) and compare the outcomes to those of conventional THA. **Methods:** This retrospective cohort analysis of the ACS-NSQIP database queried data on THA patients (CPT 27130) from 2015 to 2020. Technology assistance was identified with CPT 20985, 0054T, and 0055T. Matched cohorts were created to compare clinical comorbidities and adverse events. **Results:** This analysis included 219,216 conventional THAs and 2258 cases utilizing TA-THA. The number and percentage of surgeries utilizing technology, as well as the average operative time, consistently rose from 2015 to 2019, with all declining in 2020. Length of stay decreased yearly from 2015 to 2019, with an increase in 2020. There were no significant differences in the incidence of adverse events by year. Matched cohort analysis demonstrated that TA-THA led to longer operative times (102.6 ± 35.6 vs. 91.6 ± 37.4 min, *p* < 0.001) and a shorter average length of stay (1.6 ± 1.4 vs. 2.0 ± 1.9 days, *p* < 0.001). Transfusion rates were higher in the TA-THA cohort (6.0% vs. 4.4%, *p* = 0.013). **Conclusions:** The usage of TA-THA increased from 2015 to 2019, with declines during 2020. TA-THA led to longer operative times, increased transfusion rates, and no difference in the incidence of adverse events compared to conventional arthroplasty. These findings demonstrate that TA-THA is growing in popularity without a significant improvement in short-term complication rates.

## 1. Introduction

Hip osteoarthritis is the second most common form of osteoarthritis and one of the leading causes of age-related decline in mobility and function, with radiographic evidence present in nearly 20% of individuals over 50 [1,2]. Total hip arthroplasty (THA) is regarded as one of the most successful orthopedic procedures, with consistent clinical and patient-reported outcomes [3]. The first generation of robotic arthroplasty was introduced in the 1990s to improve implant placement [4]. Many of the newer systems have the ability to incorporate preoperative CT imaging into the surgical plan and provide a haptic window that restricts the area in which surgeons are allowed to operate [5]. Alternatively, others forego the need for preoperative imaging and rely on their skillset to properly orient the implants during surgery.

With the potential for improving accuracy and precision in surgical techniques, there is significant interest in the role of robotics and technology assistance. Trends show increased prevalence among hospitals and utilization among arthroplasty surgeons, with the percentage of cases utilizing technology increasing from 0.1% to 1.9% from 2005 to 2018 and the total number of technology-assisted cases nearly tripling from 2008 to 2015 [6,7]; however, controversy remains regarding the clinical and cost benefits of implementing the technology. Technology-assisted arthroplasty has demonstrated superior consistency and accuracy in many measures of implant orientation at the expense of longer operative times [8,9]. However, many analyses have found that improved implant alignment does not directly translate to better functional outcomes or fewer complications [10,11]. Some argue against the implementation of technology due to its higher upfront and perioperative costs, though others have found that total spending is less when including the 90-day postoperative period [12]. This may be in part due to patient selection; after surgery, robotic-assisted patients were less likely to stay in a skilled nursing facility and were more likely to have home health services [13].

To justify the costs associated with maintaining and developing robotic technology, it is essential to assess its impact on clinical outcomes. This study aims to analyze six years of nationwide trends in the utilization, patient demographics, clinical variables, and outcomes of technology assistance in primary THA using data from the American College of Surgeons National Surgical Quality Improvement Program (ACS-NSQIP) database. Additionally, we created demographic- and comorbidity-matched cohorts of 2258 conventional and technology-assisted THA patients to compare clinical variables and the incidence of adverse events to evaluate the proficiency and benefits of the use of assistive technology in THA.

## 2. Materials and Methods

The American College of Surgeons National Surgical Quality Improvement Program (ACS-NSQIP) was used to conduct this analysis. NSQIP data were collected by the certified Surgical Clinical Reviewer at each participating site. Participant Use Data Files (PUF) from 2015 to 2020 were used in this analysis, which included patients who underwent surgery from January 2015 to December 2020. NSQIP uses a systematic sampling process to determine which cases are included in the PUF. All patients were followed for 30 days postoperatively.

The inclusion criteria for this analysis were any patients that underwent THA, defined by Current Procedural Terminology (CPT) code 27130. NSQIP criteria for case exclusion included minors (patients less than 18 years of age), trauma cases, and cases that were returned to the operating room due to a complication from a prior procedure. The data were cleaned for this analysis using R Studio version 2023.06.0 (Boston, MA, USA) to exclude cases that had an operative time or body mass index (BMI) less than or equal to zero. Cases were also completely excluded prior to analysis if functional status, dyspnea status, sex, or American Society of Anesthesiologists (ASA) class were unknown. If the principal anesthesia technique was “none”, “not reported”, or “other”, the case was excluded prior to analysis to ensure that this would not be a confounding variable.

The independent variable for this analysis was technology assistance during surgery, defined by procedures with CPT codes 20985, 0054T, and 0055T coded concurrently (Table 1). All patients were followed for at least 30 days following surgery. The dependent variables were the year of surgery and adverse events following surgery. The outcomes of interest were death, wound dehiscence, sepsis, pulmonary embolism, renal complication, myocardial infarction, cardiac arrest, stroke, transfusion, deep vein thrombosis (DVT), urinary tract infection (UTI), pneumonia, intubation issues, surgical site infection (SSI), and return to the operating room. Any adverse event (AAE) included all the above complications.

Technology-assisted THAs were compared by year of surgery using an ANOVA to determine if there was a difference in operative time or length of stay (LOS). An ANOVA was chosen to compare the categorical variable, year, to the continuous variables: operative time and length of stay. A chi-square test was used to determine if there was a difference in complication rates by year for technology-assisted THA. Matched cohorts for technology-assisted and conventional THA were created using 1:1 propensity score-matching to match patients according to age, BMI, sex, race, diabetes, smoking status, ASA class, use of hypertension medication, CHF, COPD, and bleeding disorder. SPSS version 28.0.1.1 (Armonk, NY, USA) was used for statistical analyses. A post-hoc Bonferroni was used to compare differences between procedure years for continuous variables. A post-hoc Bonferroni was chosen because it adjusts the *p*-value to reflect the risk of a type one error and is one of the more conservative post hoc tests. An independent sample *t*-test was used to determine if there was a difference between groups for continuous variables. A Chi-square test was used to determine if there was a difference between categorical variables. The results were considered statistically significant if *p* ≤ 0.05.

## 3. Results

### 3.1. Trends in Operative Frequency of TA-THA vs. Conventional THA

**Figure 1:**
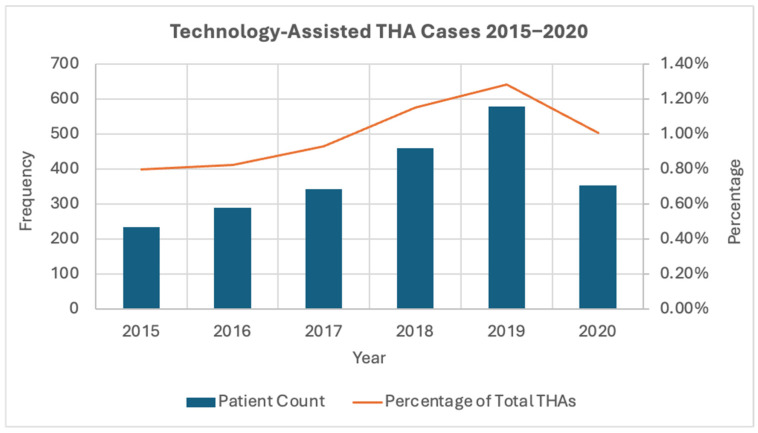
Trends from 2015 to 2020 in TA-THA patient frequency and percentage of total hip arthroplasty cases.

**Figure 2:**
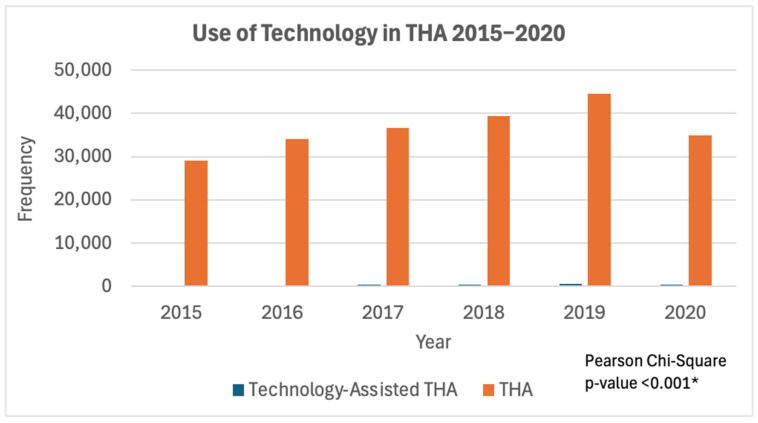
Trends from 2015 to 2020 in technology use frequency compared to conventional THA. * = Statistically significant.

This analysis included 221,474 patients within the NSQIP database who underwent total hip arthroplasty from 2015 to 2020, with 219,216 being conventional THA and 2258 being cases utilizing technology assistance. There was a consistent rise in TA-THA cases between the years of 2015 and 2019, followed by a decrease in 2020. The percentage of total cases utilizing technology assistance also increased each year from 2015 to 2019, with a peak in 2019 at 1.28%. However, the number of conventional cases consistently exceeded the number of technology-assisted cases throughout this period (*p* < 0.001). A summary of these trends comparing the incidence of technology use in THAs can be found in Figure 1 and Figure 2.

### 3.2. Demographic and Adverse Events in TA-THA

Between 2015 and 2020, there were no significant differences in the rate of postoperative adverse events for the TA-THA in the unmatched cohort (Table 2). The patient cohorts demonstrated increases in age (*p* = 0.016) and operative time (*p* = 0.001), with a decreasing LOS (*p* < 0.001) throughout this period (Table 2). There were significant differences in age between patients treated in 2015 and 2019 (63.12 ± 12.4 years vs. 65.97 ± 11.3 years; *p* = 0.022) in addition to 2015 and 2020 (63.12 ± 12.4 years vs. 66.35 ± 10.9 years, *p* = 0.014). Operative time significantly increased between 2015 and 2018 (95.97 ± 26.7 vs. 105.0 ± 33.2 min, *p* = 0.022), as well as between 2015 and 2019 (95.97 ± 26.7 min vs. 106.0 ± 36.6 min, *p* = 0.004). A significant increase in operative time was also observed between 2016 and 2019 (98.10 ± 31.1 min vs. 106.0 ± 36.6 min, *p* = 0.028). LOS significantly decreased from 2015 (2.06 ± 2.237 days) to each year between 2016 and 2020 (*p* = 0.011; 0.002; <0.001; <0.001; 0.001, respectively). The Bonferroni post-hoc analyses for age, operative time, and LOS can be found in Table 3, Table 4 and Table 5.

### 3.3. Matched Cohort Analysis of TA-THA vs. Conventional THA

Matched cohorts included 2258 patients from the conventional and technology-assisted treatment groups. They demonstrated no difference in age, BMI, sex, functional status, smoking status, ASA class, race, or comorbidities. After matching, there was a higher operative time in the TA-THA cohort compared to the conventional group (102.61 ± 35.56 min vs. 91.59 ± 37.42 min, *p* < 0.001). TA-THA patients had a shorter LOS compared to the unassisted cohort (1.64 ± 1.38 days vs. 2.03 ± 1.85 days, *p* < 0.001). Inpatient status was higher in the TA-THA cohort (93.0% vs. 91.5%, *p* = 0.052), although this failed to reach statistical significance. A summary of the matched demographic and comorbidity data can be found in Table 6.

There was no significant difference in the incidence of any adverse event within 30 days postoperatively between the matched cohorts (*p* = 0.078). Transfusion rates were higher in the TA-THA cohort than the unassisted-THA cohort (6.0% vs. 4.4%, *p* = 0.013). No other adverse event demonstrated significantly different rates between the matched groups. The incidence of adverse events in the matched cohorts can be found in Table 7.

## 4. Discussion

The purpose of this study was to analyze NSQIP data between 2015 and 2020 to investigate yearly demographic, comorbidities, and 30-day post-operative outcomes of total hip arthroplasties performed with technology assistance (TA-THA). This study comprises recent data comparing trends in TA-THA on a yearly basis to accurately portray the state of TA-THA [14], as well as comparing this period to conventional THA in order to elucidate any differences in efficacy or safety. Yearly analyses demonstrated a rise in the utilization of technology assistance for THAs between the years of 2015 and 2019, with a decline observed in 2020 for both types of hip arthroplasty. No difference was observed in yearly post-operative adverse events within TA-THA, whereas transfusion rates were lower overall in the conventional cohort. A yearly analysis of demographic and comorbidity data for technology-assisted THA determined an increase in age (2015 compared to 2019 and 2020) and operative time (2015 compared to 2018 and 2019), with a longer operative time when comparing the two types of procedures over this period. Despite an increase in operative time when comparing cohorts, LOS in TA-THA procedures was found to have declined since 2015 and was overall shorter than conventional arthroplasty throughout the total period. These findings are particularly important given the rising incidence of TA-THAs, driven by increased public interest and continued industrial growth, as well as the desire for improved clinical and functional outcomes, although the stated benefits primarily regard surgical precision.

### 4.1. Trends in Operative Frequency

The increased utilization of technology for hip arthroplasty is consistent with trends observed in previous studies [7,15], while similar findings are being described across specialties like general surgery, gynecology, and urology [16,17]. A contributing factor within orthopedics could be the improved surgical precision due to the continued industrial development of robotic systems regarding the accuracy of implantation, leg-length discrepancies, and post-implant stability [18,19]. Meanwhile, the decline observed in 2020 for both TA-THAs and conventional THAs could be explained by the impact of the COVID-19 pandemic on elective procedures within orthopedics, as well as across all specialties [20,21].

### 4.2. Comorbidities and Post-Operative Adverse Events

The drawbacks of technology-assisted arthroplasty are primarily stated to be the cost and radiologic exposure. Studies have described no significant difference when discussing clinical and functional outcomes following surgery [22]. Our study is consistent with these findings for post-operative adverse events when comparing cohorts, excluding transfusion rates. The same results were also found following a comparison of technology-assisted post-operative outcomes by year, indicating that technology-assisted hip arthroplasties provide no significant changes in safety.

The rising incidence of TA-THA despite the equivocal or non-significant improvement in functional or clinical outcomes is also compounded by an increase in public interest in technology-assisted arthroplasties over the past decade [23]. These trends require careful provider navigation in the management of patient expectations, especially when outcomes are still a matter of debate.

### 4.3. Operative Time and Length of Stay

Longer operative times are associated with increased odds of short-term complications and re-operation rates, as well increases in the costs related to the time spent in the operative room and indirect costs related to prolonged patient care [24,25,26]. While, generally, operative times for hip arthroplasties have remained stable since 2000, various studies support our finding of a significantly longer operative time for technology-assisted arthroplasty [14,19,27]. This is likely due to an increased learning curve with the use of the aforementioned technologies, as well as the required setup, which is greater than that of conventional THA. However, previous studies have additionally shown reductions in operative time as surgeons progress through training [4].

Despite an increase in operative time when comparing cohorts, LOS was found to be significantly lower in technology-assisted THA procedures than conventional procedures, consistent with previous studies [22]. Likewise, the yearly analyses displayed similar trends to those described by Hsiue et al. (3.9 days in 2005 vs. 2.5 days in 2014), with a significant reduction in length of stay past 2015 [15]. These findings warrant further exploration, as the previous literature regarding elective procedures has suggested increased operative time, ASA class, and age as significant correlators of a longer LOS [28]. This contradicts the increase in operative time and age when using technology-assisted THA observed in our yearly analysis, as well as our observation of no significant difference in ASA class for both yearly and matched cohort comparisons. In addition, we found inpatient status to be higher in the TA-THA cohort, which would further contradict the findings of a decreased LOS, although this finding failed to reach statistical significance (*p* = 0.052).

This paradoxical finding may be attributed to the more informed preoperative planning and precise surgical movements facilitated by technology assistance, which collectively enhance implant stability. Given that improper alignment poses risks of implant loosening, rejection, or infection, patients consequently experience shorter hospital stays [18,29]. As the setup required during the surgery inherently increases the operative time, future initiatives could be aimed at education and training to mitigate the learning curve associated with this procedure.

### 4.4. Transfusion Rates

Although our study found higher transfusion rates in the TA-THA cohort, there are discrepancies in the literature regarding intraoperative blood loss for hip arthroplasty. Bukowski et al. and Fontalis et al. both reported reduced or equivalent intra-operative blood loss for TA-THAs, whereas Bargar et al. and Siebel et al. each noted increased blood loss [4,30,31,32]. However, this discrepancy could be due to the earlier data used in the latter studies and the reliance on the ROBODOC system, which was largely replaced by MAKO systems in the 2010s. Likewise, the increased operative time seen in TA-THA could be a potential reason for the increased blood loss and higher transfusion rates.

### 4.5. Limitations

Limitations include this study being retrospective in nature, as well as the analysis being confined to the outcomes reported by the NSQIP database. Likewise, the large consortium of hospitals incorporated within the database leads to heterogeneity in the data provided for the study. Possible confounders include undocumented differences in the experience of the primary surgeon, which influences operative time and complication rates, as well as the robotic system and implant type utilized. Additionally, the lack of availability of pre-operative patient data, such as a history of present illness or prior management, and post-operative discrepancies in rehabilitation protocols and in-patient care by institution, could further confound claims about risk factors and associated clinical outcomes. Furthermore, limitations in the data reported for outcomes, as well as their confinement to 30 days post-operatively, provide a gap in the knowledge in an area that is crucial to the ongoing debate regarding comparisons to conventional surgery. Likewise, as this analysis is dependent on accurate coding by physicians, discrepancies in the consistency of the CPT code used for computer-navigation would result in missing patients in the technology cohort due to incomplete coding.

Future retrospective studies with a high-quality chart review that investigate these operative and post-operative differences would assist in establishing meaningful differences between operation types, while controlled prospective studies with matched surgeon experience and consistent CPT coding could provide meaningful data regarding functional and clinical outcomes.

## 5. Conclusions

This study analyzes six years of contemporary data from NSQIP to investigate yearly trends in the demographic, comorbidity, and 30-day outcomes of TA-THA, as well as utilizing matched cohorts over this period to determine differences between TA-THA and conventional hip arthroplasties. Yearly analysis demonstrated a rising incidence and percentage of hip arthroplasties with technology assistance between 2015 and 2020, as well as a higher operative time and age for these arthroplasties, despite a shorter LOS. After matching, TA-THA over this period demonstrated a longer surgical time and increased transfusion rates, with a shorter LOS when compared to the conventional THA cohort. The findings of this study largely support the recent literature, whereas discrepancies continue to exist regarding the transfusion rates between techniques. Identifying changes in the efficacy and safety of TA-THAs, as well as differences between technology-assisted and conventional surgeries, can provide crucial data for patients and physicians alike to guide clinical decision-making and operative management for end-stage hip arthritis.

## Figures and Tables

**Table 1:** CPT codes defining technology-assisted total hip arthroplasty.

CPT Code	Description
20985	Computer-assisted surgical navigational procedure for musculoskeletal procedures; imageless.
0054T	Computer-assisted musculoskeletal surgical navigational orthopedic procedure, with image guidance based on fluoroscopic images.
0055T	Computer-assisted musculoskeletal surgical navigational orthopedic procedure, with image guidance based on CT/MRI images.

**Table 2:** Yearly demographic and adverse event data in patients undergoing TA-THA (*n* = 2258).

	2015 (%)	2016 (%)	2017 (%)	2018 (%)	2019 (%)	2020 (%)	*p*-Value
Patients, *n* (%)	235 (10.4)	289 (12.8)	343 (15.2)	459 (20.3)	578 (25.6)	354 (15.7)	
Age (mean ± SD, years)	63.12 ± 12.4	64.74 ± 11.9	65.61 ± 11.6	65.55 ± 11.6	65.97 ± 11.3	66.35 ± 10.9	0.016 *
BMI (mean ± SD, kg/m^2^)	30.04 ± 5.70	30.35 ± 6.12	30.51 ± 5.82	31.03 ± 5.66	30.59 ± 5.98	30.27 ± 6.18	0.314
Operative time (mean ± SD, min)	95.97 ± 26.7	98.10 ± 31.1	102.6 ± 38.5	105.0 ± 33.2	106.0 ± 36.6	102.0 ± 41.2	0.001 *
LOS (mean ± SD, days)	2.06 ± 2.237	1.65 ± 1.563	1.62 ± 1.061	1.57 ± 1.052	1.55 ± 1.154	1.60 ± 1.403	<0.001 *
ASA class (mean ± SD)	2.55 ± 0.614	2.51 ± 0.578	2.47 ± 0.586	2.51 ± 0.538	2.49 ± 0.553	2.43 ± 0.589	0.146
Post-op adverse events	23 (9.8)	30 (10.4)	40 (11.7)	42 (9.2)	54 (9.3)	32 (9.0)	0.843
30-day reoperation	7 (3.0)	7 (2.4)	12 (3.5)	10 (2.2)	10 (1.7)	6 (1.7)	0.531
Death	0	0	2 (0.6)	1 (0.2)	1 (0.2)	0	0.441
Wound dehiscence	1 (0.4)	0	1 (0.3)	0	1 (0.2)	1 (0.3)	0.758
Sepsis	1 (0.4)	2 (0.7)	2 (0.6)	3 (0.7)	0	2 (0.6)	0.591
Pulmonary embolism	0	1 (0.3)	1 (0.3)	1 (0.2)	4 (0.7)	1 (0.3)	0.694
Renal complication	0	0	0	1 (0.2)	0	1 (0.3)	0.600
MI	1 (0.4)	2 (0.7)	0	1 (0.2)	0	1 (0.3)	0.364
Cardiac arrest	0	0	0	1 (0.2)	1 (0.2)	0	0.789
Stroke	0	0	1 (0.3)	1 (0.2)	0	0	0.585
Transfusion	12 (5.1)	16 (5.5)	27 (7.9)	28 (6.1)	35 (6.1)	18 (5.1)	0.685
DVT	2 (0.9)	1 (0.3)	1 (0.3)	1 (0.2)	4 (0.7)	2 (0.6)	0.812
UTI	1 (0.4)	2 (0.7)	3 (0.9)	4 (0.9)	5 (0.9)	3 (0.8)	0.990
Pneumonia	0	0	0	1 (0.2)	2 (0.3)	1 (0.3)	0.741
Intubation issues	0	1 (0.3)	0	1 (0.2)	0	0	0.497
SSI	2 (0.9)	2 (0.7)	5 (1.5)	6 (1.3)	6 (1.0)	4 (1.1)	0.947

* = Statistically significant.

**Table 3:** TA-THA age Bonferroni post-hoc analysis *p*-values.

	2015	2016	2017	2018	2019	2020
2015		1.000	0.164	0.134	0.022 *	0.014 *
2016			1.000	1.000	1.000	1.000
2017				1.000	1.000	1.000
2018					1.000	1.000
2019						1.000

* = Statistically significant.

**Table 4:** TA-THA operative time Bonferroni post-hoc analysis *p*-values.

	2015	2016	2017	2018	2019	2020
2015		1.000	0.410	0.022	0.004 *	0.656
2016			1.000	0.141	0.028 *	1.000
2017				1.000	1.000	1.000
2018					1.000	1.000
2019						1.000

* = Statistically significant.

**Table 5:** TA-THA LOS Bonferroni post-hoc analysis *p*-values.

	2015	2016	2017	2018	2019	2020
2015		0.011 *	0.002 *	<0.001 *	<0.001 *	0.001 *
2016			1.000	1.000	1.000	1.000
2017				1.000	1.000	1.000
2018					1.000	1.000
2019						1.000

* = Statistically significant.

**Table 6:** Demographic and comorbidity characteristics in patients undergoing TA-THA vs. conventional THA.

	Conventional THA Matched (%)	TA-THA Matched (%)	*p*-Value
Patients, *n* (%)	2258 (50.0)	2258 (50.0)	-
Age (mean ± SD, years)	65.50 ± 11.502	65.43 ± 11.585	0.839
BMI (mean ± SD, kg/m^2^)	30.51 ± 6.313	30.52 ± 5.917	0.939
Male sex (%)	975 (43.2)	991 (43.9)	0.631
Operative time (mean ± SD, min)	91.59 ± 37.424	102.61 ± 35.555	<0.001 *
LOS (days)	2.03 ± 1.850	1.64 ± 1.377	<0.001 *
Inpatient status (%)	2065 (91.5)	2100 (93.0)	0.052
Dependent functional status (%, partial or total)	40 (1.8)	58 (2.6)	0.070
Current smoker (%)	286 (12.7)	297 (13.2)	0.625
ASA Class, *n* (%)			0.795
1—No disturbance	56 (2.5)	47 (2.1)	
2—Mild disturbance	1094 (48.4)	1098 (48.6)	
3—Severe disturbance	1065 (47.2)	1074 (47.6)	
4—Life-threatening disturbance	43 (1.9)	39 (1.7)	
5—Moribund	0	0	
Race, *n* (%)			0.989
White	1812 (80.2)	1827 (80.9)	
Black	326 (14.4)	217 (14.0)	
Asian	27 (1.2)	24 (1.1)	
American Indian or Alaska Native	7 (0.3)	6 (0.3)	
Native Hawaiian or Pacific Islander	6 (0.3)	7 (0.3)	
Unknown/not reported	80 (3.5)	77 (3.4)	
Comorbidities, *n* (%)			
CHF	4 (0.2)	4 (0.2)	1.000
Renal failure	1 (0.0)	1 (0.0)	1.000
Dialysis	6 (0.3)	6 (0.3)	1.000
Steroid use	83 (3.7)	94 (4.2)	0.399
Malnourishment	6 (0.3)	7 (0.3)	0.781
Bleeding disorder	35 (1.6)	35 (1.6)	1.000
Ascites	0	0	
Pre-operative transfusion	3 (0.1)	3 (0.1)	1.000
Diabetes	304 (13.4)	312 (13.8)	0.917
IDDM	62 (2.7)	66 (2.9)	
NIDDM	242 (10.7)	246 (10.9)	
DOE	99 (4.4)	97 (4.3)	0.364
COPD	56 (2.5)	63 (2.8)	0.515

* = Statistically significant.

**Table 7:** Incidence of adverse events in patients undergoing TA-THA vs. THA (*n* = 4516).

	THA		TA-THA		*p*-Value
	No.	Rate (%)	No.	Rate (%)	
Any adverse event	187	8.3	221	9.8	0.078
Death	5	0.2	4	0.2	0.739
Wound dehiscence	6	0.3	4	0.2	0.527
Sepsis	8	0.4	10	0.4	0.637
Pulmonary embolism	9	0.4	8	0.4	0.808
Renal complication	0	0.0	2	0.1	0.157
MI	7	0.3	5	0.2	0.563
Cardiac arrest	3	0.1	2	0.1	0.655
Stroke	2	0.1	2	0.1	1.000
Transfusion	99	4.4	136	6.0	0.013 *
DVT	15	0.7	11	0.5	0.431
UTI	19	0.8	18	0.8	0.869
Pneumonia	4	0.2	4	0.2	1.000
Intubation issues	1	0.0	2	0.1	0.564
SSI	25	1.1	25	1.1	1.000
Return to OR	40	1.8	52	2.3	0.206

* = Statistically significant.

## Data Availability

The datasets used and/or analyzed during the current study are available from the corresponding author on reasonable request.
